# *Yersinia *outer protein YopE affects the actin cytoskeleton in *Dictyostelium discoideum *through targeting of multiple Rho family GTPases

**DOI:** 10.1186/1471-2180-9-138

**Published:** 2009-07-14

**Authors:** Georgia Vlahou, Oxana Schmidt, Bettina Wagner, Handan Uenlue, Petra Dersch, Francisco Rivero, Barbara A Weissenmayer

**Affiliations:** 1 Zentrum für Biochemie und Zentrum für Molekulare Medizin, Medizinische Fakultät, Universität Köln, Joseph-Stelzmann-Strasse 52, 50931 Köln, Germany; 2 Institut für Biologie – Mikrobiologie, Fachbereich Biologie, Chemie, Pharmazie, Freie Universität Berlin, Königin-Luise-Strasse 12-16, 14195 Berlin, Germany; 3 Helmholtz-Zentrum für Infektionsforschung, Inhoffentstr. 7, 38124 Braunschweig, Germany; 4 Centre for Biomedical Research, The Hull York Medical School and Department of Biological Sciences, University of Hull, Cottingham Road, Hull, HU6 7RX, UK; 5 UCD Conway Institute of Biomolecular and Biomedical Research, University College Dublin, Belfield, Dublin 4, Ireland

## Abstract

**Background:**

All human pathogenic *Yersinia *species share a virulence-associated type III secretion system that translocates *Yersinia *effector proteins into host cells to counteract infection-induced signaling responses and prevent phagocytosis. *Dictyostelium discoideum *has been recently used to study the effects of bacterial virulence factors produced by internalized pathogens. In this study we explored the potential of *Dictyostelium *as model organism for analyzing the effects of ectopically expressed Yersinia outer proteins (Yops).

**Results:**

The *Yersinia pseudotuberculosis *virulence factors YopE, YopH, YopM and YopJ were expressed *de novo *within *Dictyostelium *and their effects on growth in axenic medium and on bacterial lawns were analyzed. No severe effect was observed for YopH, YopJ and YopM, but expression of YopE, which is a GTPase activating protein for Rho GTPases, was found to be highly detrimental. GFP-tagged YopE expressing cells had less conspicuous cortical actin accumulation and decreased amounts of F-actin. The actin polymerization response upon cAMP stimulation was impaired, although chemotaxis was unaffected. YopE also caused reduced uptake of yeast particles. These alterations are probably due to impaired Rac1 activation. We also found that YopE predominantly associates with intracellular membranes including the Golgi apparatus and inhibits the function of moderately overexpressed RacH.

**Conclusion:**

The phenotype elicited by YopE in *Dictyostelium *can be explained, at least in part, by inactivation of one or more Rho family GTPases. It further demonstrates that the social amoeba *Dictyostelium discoideum *can be used as an efficient and easy-to-handle model organism in order to analyze the function of a translocated GAP protein of a human pathogen.

## Background

In the genus *Yersinia *there are three pathogenic species that can cause different diseases such as bubonic plague or gastrointestinal disorders. *Yersinia enterocolitica *is an important human pathogen that can also provoke a variety of extraintestinal clinical syndromes, e. g. systemic arthritis. The main strategy used by *Yersinia *to overcome the host immune system is the blockage of phagocytosis by cells of the innate immune system and the silencing of inflammatory reactions [1]. For this purpose *Yersinia *translocates at least six so-called *Yersinia *Outer Proteins (Yops) into the host cell via a type III secretion system [2,3]. The Yop effector proteins interfere with different eukaryotic cell signaling pathways and/or disrupt the cytoskeleton in a specialized way. For example, YopH is a phosphotyrosine phosphatase that inactivates components of focal adhesion complexes in mammalian cells [4] and induces apoptosis of infected T cells [5]. Two other Yop effectors, YopJ/P and YopM, affect components of signal transduction pathways in the cytosol or nucleus. YopJ is a cysteine protease that inhibits MAPK and NF-κB signaling pathways and promotes apoptosis in macrophages [6,7]. YopM consists mainly of leucine rich repeats, accumulates in the nucleus and has apparently no enzymatic activity [8].

Another *Yersinia *effector protein attacking the mammalian cell cytoskeleton is YopE. In cooperation with other Yops YopE disrupts the actin cytoskeleton [9-12], blocks phagocytosis [9,12,13] and inhibits inflammatory responses [14-16]. In vitro, YopE is a GTPase activating protein (GAP) for RhoA, Rac1 and Cdc42 although the substrate specificity may differ inside the cell [10-12,17-19]. More recently YopE has been found to inactivate also RhoG [20]. Infection studies on mice have shown that YopE is a very important virulence factor for the pathogenesis of all pathogenic *Yersinia *[21]. YopE is targeted to a perinuclear compartment recently identified as the Golgi apparatus and the endoplasmic reticulum, and this localization appears to be an important factor determining the substrate specificity for the GTPases [20,22].

Studies in which Yops have been ectopically expressed in mammalian cells [3] or, less frequently, yeast cells [10,23] have proved useful to understand the roles of these effectors. More recently the social amoeba *Dictyostelium discoideum *has been found helpful for the analysis of bacterial virulence factors as has been shown for *Legionella pneumophila*, *Pseudomonas aeruginosa*, *Mycobacterium *spp. and *Vibrio cholerae *[24]. The advantage of the social amoeba as a new host model organism for bacterial pathogenicity lies in its ability to phagocytose, which brings *Dictyostelium *in close relationship to professional mammalian phagocytes [25]. The structural and regulatory components necessary for the rearrangement of the cytoskeleton during phagocytosis are highly conserved from simple eukaryotes to man [26,27]. As the cytoskeleton is one of the major targets of pathogens, *Dictyostelium *appears as a suitable alternative for the analysis of cellular aspects of pathogenesis. *Dictyostelium* is genetically tractable, its genome is sequenced and a well characterized collection of cytoskeleton and signaling mutants are available [26], and host determinants of susceptibility and resistance to infections can easily be identified [28]. A considerable advantage of *Dictyostelium *over mammalian cell cultures is the fact that it is easy to cultivate, as the cells grow in inexpensive media without the need for a CO_2 _atmosphere.

We investigated whether *Dictyostelium* is a suitable model for translocated Yersinia effector proteins by expressing YopE, YopH, YopJ and YopM of Y. pseudotuberculosis and measuring their effects on vegetative growth. YopE, which appeared to be largely membrane-associated, proved to be highly toxic for *Dictyostelium*. We therefore examined the influence of YopE on phagocytosis, F-actin content and distribution, actin polymerization response after cAMP stimulation, and chemotaxis. The phenotype elicited by YopE in *Dictyostelium* can be explained, at least in part, by inactivation of one or more Rho family GTPases. Because YopE appears to affect pathways conserved from amoeba to man, *Dictyostelium* constitutes an appropriate model to study virulence factors that target structural and regulatory components of the actin cytoskeleton.

## Results

### Expression kinetics of *Yersinia *Yop effectors in *Dictyostelium *with an inducible Tet-off vector system

**Figure 1:**
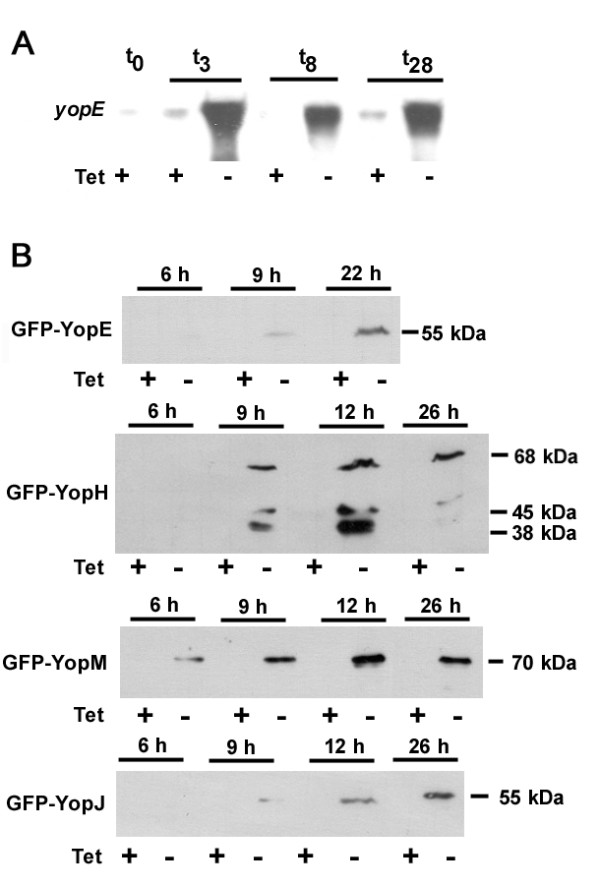
**Kinetics of Yop expression in *D. discoideum***. (A) Expression of *yopE *was induced by removal of tetracycline (-Tet). At indicated time points (in hours), total RNA of 10^7 ^cells was separated on 1.2% agarose/6.6% formaldehyde gels, transferred onto a nylon membrane, and probed with DIG-labeled *yopE*. (B) Expression of GFP-Yop fusion proteins. Expression was induced by removal of tetracycline (-Tet). At indicated time points (in hours), total cell protein from 5 × 10^5 ^vegetative cells was separated on 15%polyacrylamide/0.1% SDS gels and blotted onto nitrocellulose. Blots were probed with a GFP-specific antibody.

In order to study the effects of *Yersinia *virulence factors on *Dictyostelium *we expressed YopE, YopH, YopJ and YopM with an inducible vector system regulated by tetracycline [29]. The *yopE*, *yopH*, *yopJ*, and *yopM *genes of *Y. pseudotuberculosis *were cloned as *gfp*-fusion constructs or single genes in the tetracycline responsive vector pMB38 and expression over time was analyzed on Northern and Western blots. Fig. 1A shows that transcription of *yopE *was strongly induced 3 hours after removal of tetracycline and remained at higher levels even after 28 hours. A very small amount of the *yopE *mRNA was also detectable in the presence of tetracycline, indicating that the promoter is not completely off under non-inducing conditions. Very similar results were obtained for expression of *yopH *in this system (not shown). Synthesis of all N-terminal tagged GFP-Yop fusion proteins was observed after 6–9 hours and maximum protein expression was found between 12–26 hours (Fig. 1B). Only GFP-YopH was partially degraded, whereas all other fusion proteins appeared stable. In contrast, no expression of any of the proteins was detectable in the presence of tetracycline.

### YopE inhibts growth of *Dictyostelium*

**Figure 2:**
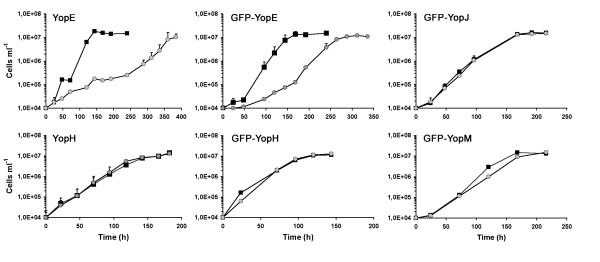
**YopE inhibits amoebial growth**. Vegetative growth was measured in liquid cultures of cell lines with non-induced and induced expression of YopE, GFP-YopE, YopH, GFP-YopH, GFP-YopJ and GFP-YopM. Black squares: non-induced cell lines; grey circles: induced cell lines. For each growth curve, two independent cultures, each run in duplicate, were analyzed and averaged. Standard error bars are mostly smaller than symbol sizes.

First we tested whether growth of *Dictyostelium *in liquid culture was affected by* in vivo *expression of Yop effectors. Growth measurements over several days showed that the growth of YopE and GFP-YopE expressing cell lines was drastically reduced in comparison with non-induced cell lines (Fig. 2). At the beginning, growth of YopE expressing cells was significantly reduced, with generation times of about 62 hours in comparison with 12 hours of the non-induced controls. After 10 days, the cells of the same culture started to regrow, albeit slower than the control cells with generation times of 20 and 38 hours. Unlike YopE, growth of *Dictyostelium *cell lines expressing other Yops or their GFP-fusion derivatives showed no noticeable difference between induced and non-induced cell lines (Fig. 2). Comparable results were obtained when the cells were plated on *Klebsiella *lawns and the plaque numbers were counted after 4 days. Only the plaque numbers of YopE or GFP-YopE expressing cell lines were reduced in comparison with the non-induced cell line (not shown).

We next investigated whether the growth defect of GFP-YopE expressing cells is due to a defect in cell division. However, DAPI staining of GFP-YopE expressing cells showed no alteration of the distribution of nuclei numbers compared to the non-induced cells, irrespective of whether cells were grown in suspension or on substrate (data not shown). In both conditions most of the cells of all cell lines were mononucleated (60–80%), the rest remained mainly binucleated.

### YopE associates with intracellular membranes

**Figure 3:**
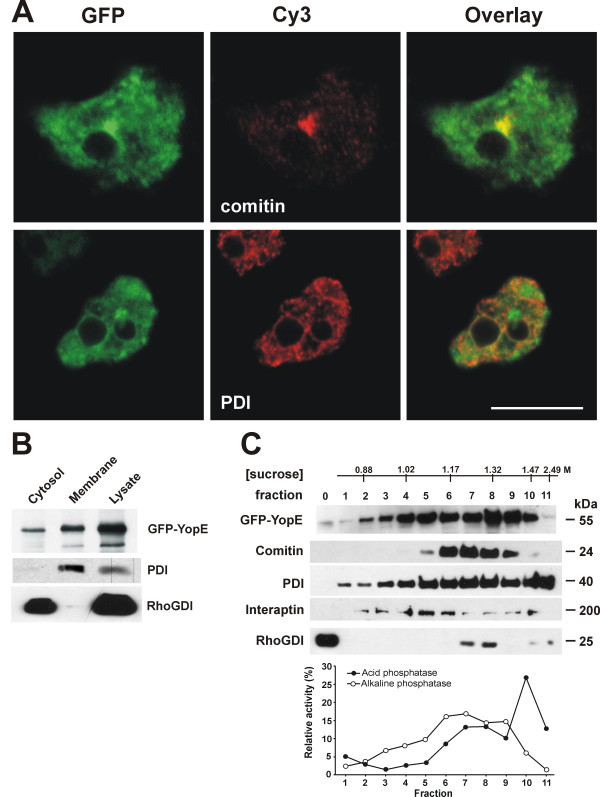
**YopE associates with intracellular membrane compartments**. (A) YopE colocalizes with markers of intracellular membrane compartments. Cells expressing GFP-YopE were fixed in cold methanol and were incubated with monoclonal antibodies that recognize the Golgi marker comitin and the ER marker protein disulfide isomerase (PDI) followed by incubation with Cy3-labeled anti-mouse IgG. GFP is visualized directly. Images are confocal sections. Scale bar, 10 μm. (B) Fractionation of *Dictyostelium *cells expressing GFP-YopE. Cells were lysed by sonication and cytosolic and membrane fractions were separated by ultracentrifugation. Samples were resolved in 12% polyacrylamide gels, blotted onto nitrocellulose membranes and probed with antibodies against GFP, PDI (marker for the membrane fraction) and RhoGDI (marker for the cytososlic fraction). (C) Sucrose gradient fractionation of cells expressing GFP-YopE. Fractions were collected from the top and analyzed in Western blots using antibodies for the indicated proteins or in enzymatic reactions. Interaptin is a protein of the nuclear envelope and ER. RhoGDI is a predominantly cytosolic protein but a small amount appears associated to membrane compartments. Alkaline phosphatase is a marker for plasma membrane and the contractile vacuole and acid phosphatase is a marker for lysosomes.

Because YopE was the only effector eliciting alterations in *Dictyostelium*, we analyzed the YopE expressing strain in more detail. From YopE it was known that it localizes at the perinuclear membrane of mammalian cells [20,22]. In *Dictyostelium *GFP-YopE appears to associate with intracellular membranes, particularly with the Golgi apparatus and less conspicuously with the endoplasmic reticulum (ER), as shown by immunofluorescence using the Golgi marker comitin and the ER marker protein disulfide isomerase (Fig. 3A). An association of YopE with other membrane compartments is also possible, however colocalization with markers for other compartments, like vatA (a subunit of the vacuolar H^+^-ATPase predominantly present at the contractile vacuole and to a lesser extent at endosomes), or vacuolin (a marker of a postlysosomal compartment) was not conclusive in fixed cells (data not shown). Fractionation of the GFP-YopE expressing cells in cytosol and membranes confirmed that YopE is predominantly membrane-associated (Fig. 3B). GFP-YopE appeared broadly distributed in a discontinuous sucrose gradient of a cell lysate, indicating that the protein associates to multiple membrane compartments (Fig. 3C).

### Inhibition of phagocytosis by YopE expression

**Figure 4:**
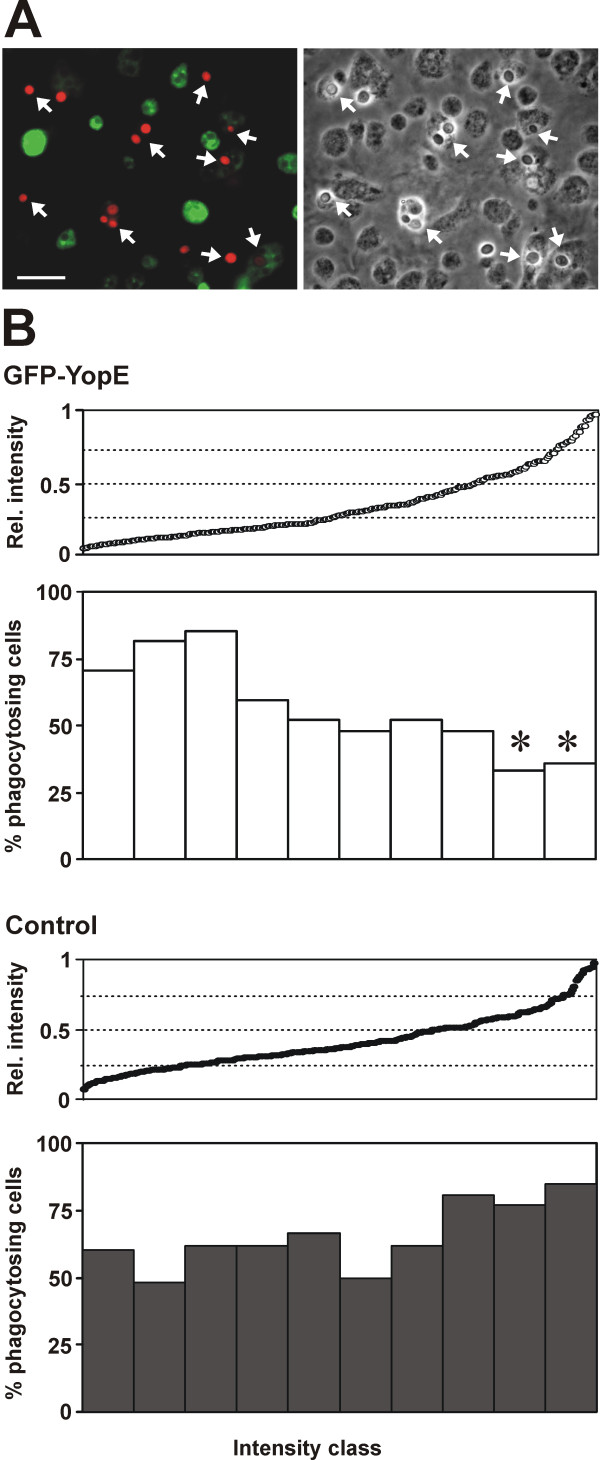
**Impaired phagocytosis in GFP-YopE expressing cells**. (A) Cells were allowed to phagocytose TRITC-labeled yeast particles on coverslips for 30 minutes before fixation. Arrows indicate yeast particles internalized by *Dictyostelium *cells. Note that cells expressing large amounts of the GFP fusion have no internalized particles. Scale bar, 25 μm. (B) Cells were treated as in A and scored for the presence of internalized particles. Control cells are cells of the parental strain MB35 expressing GFP. The intensity of GFP expression was quantitated with Image J. The diagrams display the distribution of the corresponding cell population according to the GFP levels. The populations were divided in 10 equally large classes and the proportion of phagocytosing cells was calculated. 259 control and 271 GFP-YopE cells from 4 coverslips were scored. *P < 0.05 relative to the average proportion of phagocytosing cells in the control population.

The inhibitory effect of YopE on phagocytosis is well documented in mammalian cells [9,12,13]. Because *Dictyostelium *is a professional phagocyte, we investigated this parameter in detail. We first scored individual cells fixed after exposure to fluorescently labeled yeast particles and observed that cells that express GFP-YopE have less frequently internalized yeast particles compared to cells of the same population that lack visible GFP-YopE (Fig. 4A). When we calculated uptake rates along the whole range of expression levels we observed that in the GFP-YopE strain the uptake rate roughly correlated inversely with the expression levels of the fusion protein, with strong expressors (those with relative GFP-YopE intensity over 0.5) displaying a significantly reduced uptake rate. GFP alone had no deleterious effect on the rate of particle uptake (Fig. 4B).

### YopE expression results in altered F-actin content and distribution

**Figure 5:**
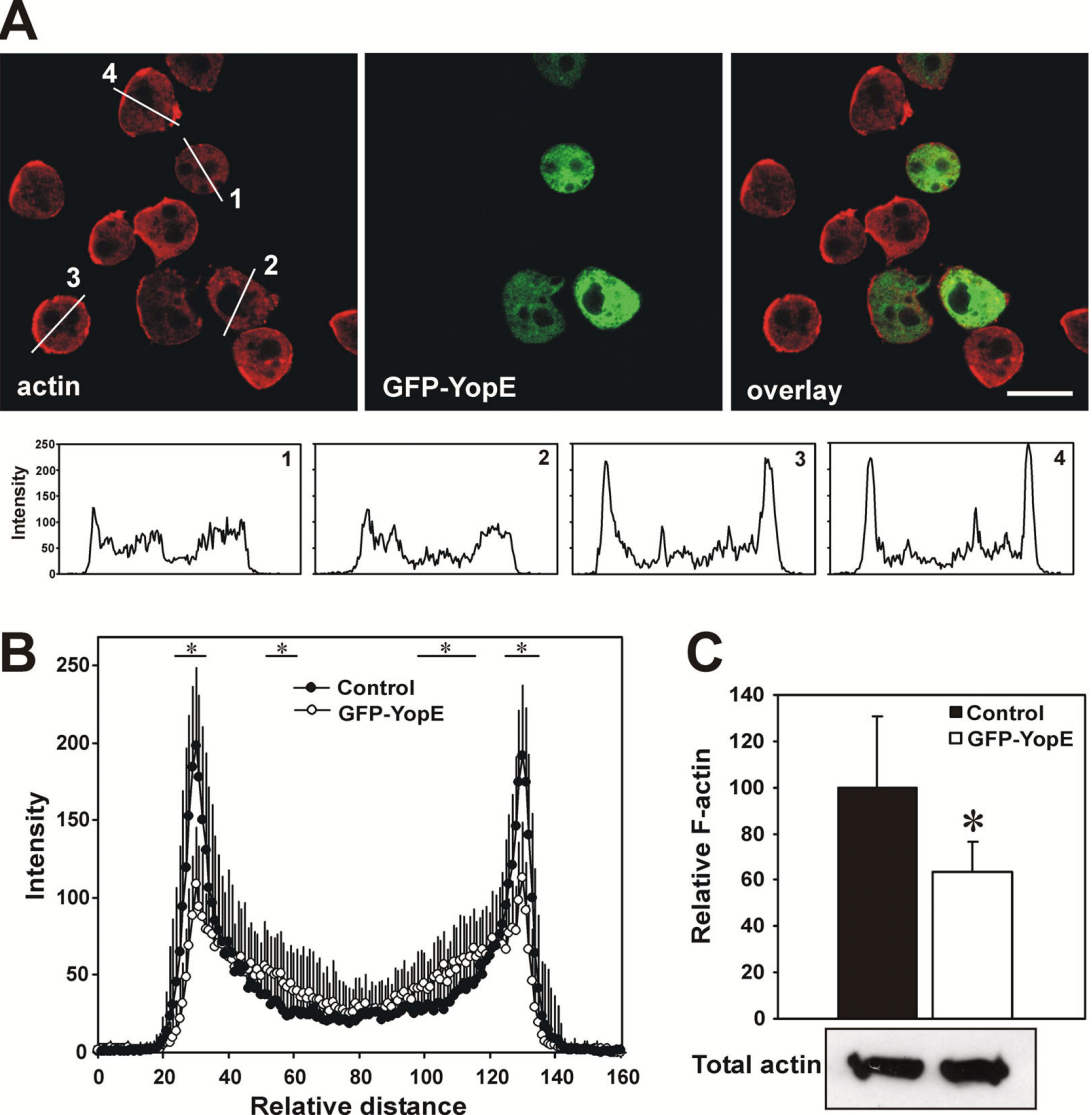
**Altered actin distribution in GFP-YopE expressing cells**. (A) Induced GFP-YopE expressing cells were allowed to sit on glass coverslips, fixed and stained with actin-specific mAb Act 1–7 followed by Cy3-labeled anti-mouse IgG. Images are confocal sections. Note that cells expressing large amounts of the GFP fusion have visibly less cortical actin. Examples of intensity profiles across cells that express large amounts GFP-YopE (1, 2) or no visible GFP-YopE (3, 4) are shown. Scale bar, 10 μm. (B) Intensity profiles across cells stained with actin-specific antibody. Control cells are induced cells that do not express GFP-YopE. The fluorescence intensity was determined for 30 cells from two independent preparations and the distance between the maxima at the cell cortex normalized. Shown is the average ± standard deviation. For simplicity, error bars are depicted in one direction only. *P < 0.05, Student's t-test. (C) Relative F-actin content of vegetative cells as determined by TRITC-phalloidin staining. Values were normalized to the total protein content of the sample. Unaltered total actin amounts were verified by Western blotting of total cell lysates. (5 μg of total protein) probed with mAb Act1-7. Control cells are non-induced cells carrying the GFP-YopE plasmid. Data are average ± standard deviation of 6 independent determinations. *P < 0.05, Student's t-test.

Because YopE is a GAP for Rho GTPases, which have been mainly implicated in regulation of actin remodeling, we investigated whether expression of YopE resulted in changes in the amount and distribution of actin. When GFP-YopE expressing cells were fixed and stained with an actin specific monoclonal antibody, we observed a weaker staining and a less conspicuous cortical accumulation of actin in cells that express GFP-YopE compared to cells of the same population that lack visible GFP-YopE (Fig. 5A). This is apparent in the intensity profiles across the cells of both populations (Fig. 5B). Quantification of F-actin levels revealed that vegetative GFP-YopE expressing cells contained significantly less F-actin (on average about 40%) than the parental strain although the total amount of actin was unaltered (Fig. 5C).

### YopE expression causes deficient actin polymerization and impaired Rac1 activation in response to cAMP

**Figure 6:**
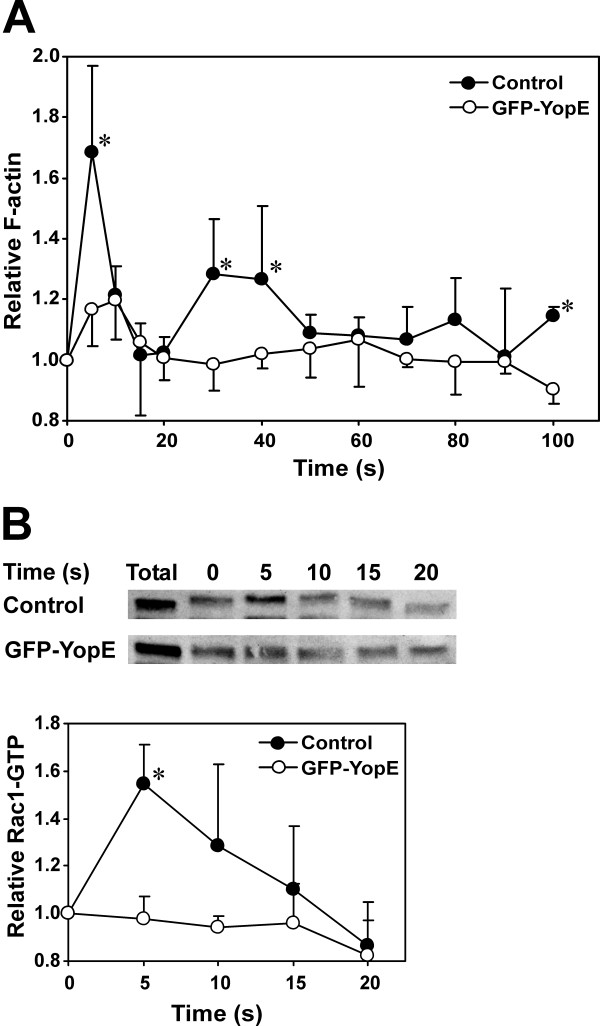
**Reduced actin polymerization response and Rac1 activation upon cAMP stimulation in YopE expressing cells**. (A) Relative F-actin content as determined by TRITC-phalloidin staining of aggregation competent cells fixed at the indicated time points after stimulation with 1 μM cAMP. Control cells are non-induced cells carrying the GFP-YopE plasmid. The amount of F-actin was normalized relative to the F-actin level of unstimulated cells. Data are average ± standard deviation of 5 independent experiments. For simplicity, error bars are depicted only in one direction. *P < 0.05, Student's t-test. (B) Activation of Rac1 upon cAMP stimulation in cells expressing GFP-YopE. Rac1-GTP was separated using a pulldown assay. A representative blot of each strain is shown. Data are average ± standard deviation of four independent pull down experiments. *P < 0.05, Student's t-test.

In *Dictyostelium *stimulation with cAMP elicits fast and highly transient changes in the F-actin content and constitutes an excellent tool to monitor alterations in the signaling pathways that regulate actin polymerization. We therefore determined the time course of actin polymerization upon cAMP stimulation in GFP-YopE expressing cells (Fig. 6A). In control cells stimulation with cAMP resulted in a rapid and transient 1.7-fold increase in the amount of F-actin followed immediately by a second lower polymerization peak that lasted until approximately 50 seconds. In contrast, GFP-YopE expressing cells showed a single, significantly lower F-actin peak (about 1.2-fold) shortly after stimulation with cAMP.

We then studied whether the altered F-actin response had an effect on the motility of the amoeba. For this, aggregation competent cells were allowed to migrate toward a micropipette filled with 0.1 mM cAMP and time-lapse image series were taken and used to generate migration paths and calculate cell motility parameters (Table 1). We found that both in the absence or presence of cAMP, GFP-YopE expressing cells and the control strain exhibited a similar behavior: cells became polarized, formed streams and migrated toward the tip of the micropipette (not shown).

**Table 1:** Analysis of cell motility of GFP-YopE cells

	Control	GFP-YopE
**Buffer**		
Speed (μm/min)	7.35 ± 3.62	7.27 ± 3.18
Persistence (μm/min × deg)	2.10 ± 1.25	2.23 ± 1.50
Directionality	0.42 ± 0.24	0.53 ± 0.25
Directional change (deg)	40.01 ± 14.51	38.41 ± 15.52
**cAMP gradient**		
Speed (μm/min)	9.02 ± 2.89	8.23 ± 3.08
Persistence (μm/min × deg)	2.94 ± 1.72*	2.83 ± 1.53
Directionality	0.78 ± 0.19*	0.71 ± 0.21*
Directional change (deg)	20.13 ± 10.49*	26.49 ± 12.69*

Time-lapse image series were captured and stored on a computer hard drive at 30 seconds intervals. The DIAS software was used to trace individual cells along image series and calculate motility parameters. Objects whose speed was <2 μm/min were excluded from the analysis. Persistence is an estimation of movement in the direction of the path. Directionality is calculated as the net path length divided by the total path length, and gives 1.0 for a straight path. Directional change represents the average change of angle between frames in the direction of movement. Values are mean ± standard deviation of approximately 50 cells from at least three independent experiments. Control cells are cells of the parental MB35 strain. * P < 0.01 relative to the same strain in buffer (Student's t test).

The actin polymerization response upon cAMP stimulation depends on the activation of Rho GTPases [30,31]. To investigate whether the alterations elicited by YopE expression result from impaired activation of Rac we used a pull-down assay to quantitate activated Rac1 upon cAMP stimulation. In control cells the chemoattractant elicited a rapid and transient increase of activated Rac1. This peak of activated Rac1 was absent in GFP-YopE expressing cells (Fig. 6B), suggesting that the defects observed in this strain are due, at least in part, to impaired Rac1 activation.

### YopE partially blocks the effects of RacH

**Figure 7:**
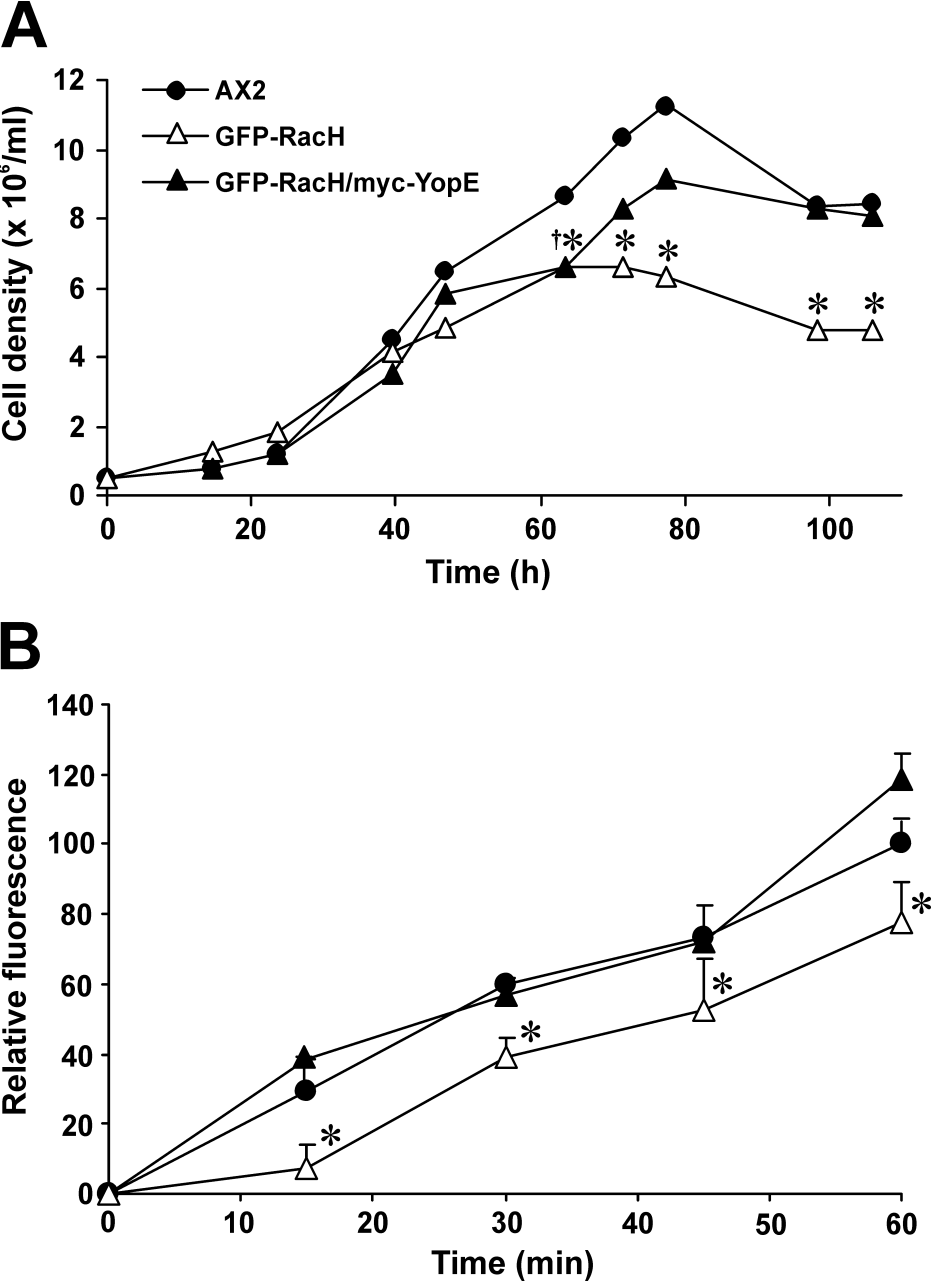
**YopE blocks the effects of RacH on growth and endocytosis**. (A) Growth in nutrient medium. Cultures were inoculated at a density of 0.5 × 10^6 ^cells/ml. The graph is representative of two independent experiments, each run in duplicate. * P < 0.05 of GFP-RacH relative to AX2, † P < 0.05 of GFP-RacH/myc-YopE relative to AX2; ANOVA. (B) Fluid-phase endocytosis of FITC-dextran. Cells were resuspended in fresh axenic medium at 5 × 10^6 ^cells/ml in the presence of 2 mg/ml FITC-dextran. Fluorescence from the internalized marker was measured at selected time points. Data are presented as relative fluorescence, AX2 being considered 100%. Four independent experiments are averaged. For clarity, error bars are depicted only in one direction. * P < 0.05 relative to AX2, ANOVA.

The spectrum of alterations elicited by YopE in *Dictyostelium *suggest that several Rho GTPases may be affected by this protein. Our attempts to determine the specificity of YopE against a panel of *Dictyostelium *GST-fused Rho GTPases in pulldown experiments were hampered by the rapid degradation of GFP-YopE upon cell lysis. The subcellular localization of YopE, in particular the association with several membrane compartments, suggested that RacH might be one of the Rho GTPases targeted by YopE. If that is the case, expression of YopE in a strain that overexpresses RacH should revert, to some extent, the defects characteristic for RacH overexpression i.e. impaired growth and reduced fluid phase uptake [32]. Because strong overexpression of RacH abolishes growth and pinocytosis, we generated a *Dictyostelium *strain that moderately overexpressed GFP-RacH. Cells of this strain displayed a growth defect in nutrient medium (Fig. 7A) and a moderate pinocytosis defect (Fig. 7B). These defects were no longer apparent when GFP-RacH and myc-tagged YopE were co-expressed, suggesting that RacH could also be a target of YopE.

## Discussion

In this study a tetracycline controlled vector system was successfully used for *de novo *expression of *Yersinia *virulence-associated Yop effector proteins in *Dictyostelium*. We found profound alterations in the amounts and localization of filamentous actin and in processes that depend on a functional actin cytoskeleton in cells expressing YopE. In contrast, expression of YopH, YopJ and YopM did not cause obvious alterations. In mammalian cells YopH silences early phagocytosis signals by dephosphorylation of components of focal adhesion complexes such as FAK, p130Cas and Fyb. The protease YopJ is known to inhibit MAPK and NF-κB pathways and to promote apoptosis [6,7]. No homologues of the focal adhesion proteins have been identified in the *Dictyostelium *genome, and a NF-κB pathway, as well as a caspase-mediated apoptosis pathway are also absent in this organism. This would explain the absence of effects of YopH and YopJ in *Dictyostelium*. Similarly, although GFP-YopM accumulated in the nucleus of *Dictyostelium *(data not shown) as in yeast and mammalian cells [8], its expression caused no measurable defects under standard growth conditions. It is possible that its targets are absent or are modified in a way that they cannot be recognized by the virulence factor in *Dictyostelium*.

YopE specifically targets the microfilament system of *Dictyostelium*, and this results in decreased basal levels of polymerized actin and less accumulation of actin at the cell cortex. The effects of YopE on the actin cytoskeleton have been widely studied in diverse mammalian cell types, like epithelial cells [33], fibroblasts [13], macrophages [34] and dendritic cells [9], where introduction of YopE causes disruption of actin filaments. YopE targets the actin cytoskeleton indirectly via modulation of small Rho GTPases, and we show that this is also the case in *Dictyostelium*. In the *Dictyostelium *genome there are no homologues of RhoA and Cdc42, but more than 18 *rac *like genes have been identified [27]. Here, we present indirect evidence showing that YopE acts on Rac1 and probably also on RacH. However, not all Rac-like proteins of *Dictyostelium *seem to be affected by the GAP activity of YopE, as the first peak of the F-actin response upon cAMP stimulation was not completely abolished and chemotaxis remained largely unaffected. This F-actin response depends mainly on RacB, RacC and Rac1 [30,35-37]. Similarly, the growth defect of YopE and GFP-YopE expressing cells is not a result of inhibited cytokinesis, suggesting that RacE [38] or other Rac proteins primarily regulating this process are not substrates of YopE.

In *Dictyostelium *YopE is predominantly membrane-associated but is not restricted to a particular compartment. It distributes rather broadly, with some enrichment at the Golgi apparatus. In mammalian cells YopE is targeted to a perinuclear membrane compartment, and residues 54–75 of YopE were sufficient for its intracellular localization [22]. More recently that compartment has been identified as the Golgi apparatus and the endoplasmic reticulum in agreement with our data in *Dictyostelium *[20,39]. It has been discussed whether the intracellular localization of YopE contributes to the substrate specificity of its GAP activity for different Rho GTPases, like Rac1 [19] and more recently RhoG [20]. As YopE overexpression reduces growth in nutrient medium and the ability of *Dictyostelium *to phagocytose it seems rather likely that it affects small GTPases implicated in endocytosis. Several Racs have been found implicated in the regulation of fluid and particle uptake in *Dictyostelium*, including Rac1, RacB RacC, RacG and RacH [31,32,36,40,41]. By virtue of its wide membrane localization YopE is therefore in a position to inactivate diverse Rac proteins in *Dictyostelium*. Notably, RacH localizes at the Golgi apparatus, ER, and the nuclear envelope [32], suggesting that YopE might counteract its function. In agreement with this, we found that YopE is able to block the effects of overexpressing RacH. It is tempting to speculate that some of the toxic effects caused by YopE in mammalian cells might be caused by inhibition of the activity of Rho family GTPases other than those that have been investigated more extensively.

## Conclusion

In mammalian cells the *Yersinia *outer membrane protein YopE has been shown to stimulate GTP hydrolysis of RhoA, Cdc42 and Rac1 resulting in disruption of the cytoskeleton and inhibition of phagocytosis. By ectopically expressing YopE in *Dictyostelium*, we show that similarly Rac1 and possibly also RacH are *in vivo *targets of this bacterial effector protein. This indicates that more GTPases might be affected by YopE, and this might depend on the intracellular localization of the virulence factor. As processes like endocytosis and actin polymerization can be analyzed in great detail, *Dictyostelium *offers a great potential for studies of phenomena at the interface of bacterial and eukaryotic interaction.

## Methods

### Plasmids and strains

*D. discoideum *AX2 and MB35, the AX2 cell line transformed with the Tet-off transactivator plasmid pMB35 [29], were used throughout the study. The open reading frames of *yopE*, *yopH*, *yopM *and *yopJ *were amplified by PCR with Ex Taq Polymerase (Takara, Gennevilliers, France) from genomic DNA of *Y. pseudotuberculosis *YPIII [42]. The PCR products were cloned in pDrive with a PCR cloning kit (Qiagen, Hilden, Germany) and subcloned in frame with the 3'-end of *gfp *in pOS8. pOS8 was constructed by PCR amplification of the *gfp *gene from pDEX-RH-*gfp *(redshiftet S65T GFP mutant from *Aequorea victoria*) [43] with the oligodeoxynucleotides 5'TGA TCA ATG AGT AAA GGA GAA GAA CTT TTC3' and 5'AGATCT GGATCC TGC ACC TGC ACC TTT GTA TAG TTC ATC CAT GCC3'. The PCR fragment was cloned in pDrive, excised with *Bgl*II and *Bcl*I and subcloned in *Bgl*II digested pMB38. For expression of a myc tag fusion *yopE *was amplified by PCR using oligodeoxynucleotide 5'GAATTC AAA ATG GAACAA AAA TTA ATT TCA GAA GAA GAT TTA ATG AAA ATA TCA TCA TTT ATT TCT ACA TC3'; which incorporates the coding sequence for the myc tag, and a specific reverse primer. The PCR fragment was cloned into pGEM-Teasy (Promega, Madison, WI, USA), excised with *Eco*RI and *Hind*III and subcloned in pDEXbsr. This vector was constructed by subcloning the blasticidin resistance cassete of pbsrΔBam [44] and the actin 8 terminator from pDEX-RH in pBluescript (Stratagene, La Jolla, CA, USA). All PCR-amplified fragments used for cloning were verified by DNA sequencing. A plasmid for expression of GFP-fused RacH has been described elsewhere [32].

### Growth of *Dictyostelium discoideum*

*D. discoideum *AX2 cells or transformants were grown at 22°C in AX medium [45]. Growth rates were determined by inoculating 10^4 ^cells/ml in 30 ml AX medium. Cells were shaken at 150 rpm and 22°C. Culture densities were monitored using a Neubauer counting chamber.

### Transformation of *Dictyostelium discoideum*

*D. discoideum *AX2 or MB35 cells were grown in AX medium to a density of 5 × 10^6 ^cells/ml, washed twice with ice-cold H-50 buffer (20 mM HEPES, 50 mM KCl, 10 mM NaCl, 1 mM MgSO_4_, 5 mM NaHCO_3_, 1 mM NaH_2_PO_4_), resuspended at 2 × 10^7 ^cells/ml, and 100 μl of this suspension was electroporated with 10 μg of plasmid DNA [46]. Transformed cells were grown on suitable selective media (ampicillin 100 μg/ml; G418 20 μg/ml; blasticidin S 10 μg/ml; tetracycline 10 μg/ml), and clonal populations were obtained by serial dilution in microtiter plates. Successful transformation of plasmids was verified by PCR or Western blot.

### Induction of Yop expression with the inducible Tet-off vector system

Induction of expression was triggered by removal of tetracycline from the medium. The cultures were washed twice with ice-cold Soerensen phosphate buffer (17 mM Na-K phosphate, pH 6.0) and inoculated to 10^4 ^cells/ml (growth measurements), or to 10^6 ^cells/ml in fresh AX medium. Induction times are indicated in each experiment.

### Plaque assays

For plaque assays, 1.5 ml Soerensen phosphate buffer, 0.1 ml *Klebsiella *overnight culture, and 200 *D. discoideum *cells in 100–200 μl Soerensen phosphate buffer were pipetted on a 1/3 SM plate (3.3 g glucose, 3.3 g bactopepton, 0.33 g yeast extract, 0.33 g MgSO_4 _× 7 H_2_O, 0.7 g KH_2_PO_4_, 0.43 g K_2_HPO_4 _× 3 H_2_O, 18 g agarose per 1 liter). The mixture was distributed homogeneously by horizontal rotation of the plates (30 times). The agar plates were dried for 2 hours and incubated at 22°C for 4 days.

### Northern blotting

Total RNA from 10^7 ^cells was isolated using the peqGold RNA pure kit (Peqlab, Erlangen, Germany), 10 μg total RNA/lane was chromatographed on 1.2% agarose gels containing 6.6% formaldehyde. Gels were blotted onto nylon membranes, hybridized with DIG-labeled cDNA probes, and stained with CDP-Star as recommended by the manufacturer (all reagents from Roche Molecular Diagnostics, Mannheim, Germany).

### Antibodies

Actin was detected using mAb Act 1–7 [47], protein disulfide isomerase using mAb 221-135-1 [48], comitin using mAb 190-340-2 [49], the VatA-subunit of the V/H^+^-ATPase using mAb 221-35-2 [50], vacuolin using mAb 221-1-1 [51], interaptin using mAb 260-60-10 [52], RhoGDI1 with mAb K8-322-2 [53], Rac1 using mAb 273-461-3 [36], myc with mAb 9E10 (Epitomics, Burlingsame, USA) and GFP with rabbit polyclonal anti-GFP (Invitrogen Karlsruhe, Germany) or mAb K3-184-2 [54].

### SDS/polyacrylamide gel electrophoresis and Western blotting

Proteins were resolved on 12.5% polyacrylamide/0.1% SDS gels, transferred to nitrocellulose membranes, and probed with the indicated primary antibodies. Primary antibodies were detected with peroxidase-coupled goat-anti-rabbit IgG (Dianova, Hamburg, Germany).

### Fluorescence microscopy

Cells were fixed in cold methanol (-20°C) followed by incubation with Cy3-labeled anti-mouse IgG. Nuclei were stained with 4',6-diamidino-2-phenylindole (DAPI, Sigma-Aldrich, Munich, Germany). Confocal images were taken with an inverted Leica TCS-SP laser-scanning microscope with a 100× HCX PL APO NA 1.40 oil immersion objective. For excitation, the 488 nm argon-ion laser line and the 543 nm HeNe laser line were used. Images were processed using the accompanying Leica software or Image J. Conventional fluorescence microscopy was performed with a Leica DMR fluorescence microscope and images were acquired with a Leica DC350FX camera (Leica, Wetzlar, Germany).

### Endocytosis assays

Phagocytosis was assayed using TRITC-labeled yeast particles and fluid-phase endocytosis was assayed using FITC-dextran as described [55]. To monitor phagocytosis after fixation cells were allowed to sit on coverslips for 15 minutes, upon which TRITC labeled yeast particles were added. Cells were allowed to phagocytose and were fixed with cold methanol after 30 minutes. Images were acquired with a conventional fluorescence microscope as indicated above. GFP expression level and particle uptake of individual cells were analyzed. Particle uptake was scored positive if the cell had internalized one or more particles. The intensity of GFP expression was quantitated using Image J.

### Chemotaxis assay

Aggregation competent cells were prepared and stimulated with a glass capillary micropipette (Femtotip, Eppendorf, Hamburg, Germany) filled with 0.1 mM cAMP [56]. Time-lapse image series were captured and stored on a computer hard drive at 30 seconds intervals with a CCD camera. The DIAS software (Soltech, Oakdale, IA, USA) was used to trace individual cells along image series and determine cell motility parameters [57].

### Subcellular fractionation

Cells were collected by centrifugation and resuspended at a density of 2 × 10^8 ^cells/ml in MES buffer (20 mM 2-[N-morpholino]ethane sulfonic acid, 1 mM EDTA, 250 mM sucrose, pH 6.5) supplemented with a protease inhibitor mixture (Roche Diagnostics, Mannheim, Germany). Cells were lysed on ice by sonication and light microscopy was performed to ensure that at least 95% of the cells were broken. Cytosolic and particulate fractions were separated by ultracentrifugation (100,000 × *g *for 30 minutes). Alternatively the cell lysate was centrifuged to equilibrium on a discontinuous sucrose gradient atop an 84% (w/v) cushion. After centrifugation fractions were collected from the top and analyzed in Western blots or used for measurement of acid and alkaline phosphatase activities as described [52].

### F-actin determination

Chemoattractant induced F-actin formation in aggregation competent cells was quantitated as described [58]. Briefly, cells were resuspended at 2 × 10^7 ^cells/ml in Soerensen buffer and starved for 6 to 8 hours. Cells were stimulated with 1 μM cAMP and 50 μl samples were taken at various time points. The reaction was terminated by addition of 450 μl stop solution (3.7% formaldehyde, 0.1% Triton X-100, 0.25 μM TRITC-phalloidin in 20 mM potassium phosphate, 10 mM PIPES, 5 mM EGTA, 2 mM MgCl_2 _pH 6.8). After staining for 1 hour, samples were centrifuged for 5 minutes at 15,000 × *g*. Pellets were extracted with 1 ml methanol for 16 hours and fluorescence (540/565 nm) was read in a PTI fluorimeter (Photon Technology Intl., Seefeld, Germany). Essentially the same procedure was used to determine the F-actin content of vegetative cells except that fluorescence values were normalized to the total protein content of the samples as determined with the method of Lowry.

### Rac1 activation assay

The Rac1 activation assay was performed as described [31]. Cells were starved for 6 to 8 hours in Soerensen buffer at a cell density of 1 × 10^7^/ml, concentrated to 4 × 10^7^/ml and stimulated with 1 μM cAMP. Aliquots were immediately removed and lysed in 5 × lysis buffer (50 mM HEPES pH 7.5, 2.5% Triton X-100, 500 mM NaCl, 100 mM MgCl_2_, 1 mM DTT) containing protease inhibitors at 4°C. The cell lysate was then mixed with glutathione-Sepharose beads previously loaded with bacterially expressed CRIB of *Dictyostelium *WASP fused to GST. After incubation and washing proteins were eluted from the beads with sample buffer and subjected to SDS-polyacrylamide gel electrophoresis and Western blot analysis with an anti-Rac1 monoclonal antibody.

## Authors' contributions

GV and OS carried out most of the experimental work. BW and HU improved some of the experimental procedures. PD participated in the design of the study. FR and BAW conceived and coordinated the study and drafted the manuscript. All authors read and approved the final manuscript.
